# Fermented Soymilk with Probiotic *Lactobacilli* and *Bifidobacterium* Strains Ameliorates Dextran-Sulfate-Sodium-Induced Colitis in Rats

**DOI:** 10.3390/nu16203478

**Published:** 2024-10-14

**Authors:** Ashwag Jaman Al Zahrani, Amal Bakr Shori, Effat Al-Judaibi

**Affiliations:** 1 Faculty of Science, Department of Biological Sciences, University of Jeddah, Jeddah 21959, Saudi Arabia; 2 Faculty of Science, Department of Biological Sciences, King Abdulaziz University, Jeddah 21589, Saudi Arabia

**Keywords:** inflammation, colitis, fermented soymilk, dextran sulfate sodium, *Lactobacilli*, *Bifidobacterium*

## Abstract

**Background**: Current treatments for inflammatory bowel disease (IBD) are relatively futile and the extended use of drugs may reduce effectiveness. Several probiotic strains have shown promise in relieving/treating IBD symptoms. **Objectives**: The current study investigated the impact of fermented soymilk with a mixture of probiotic starter cultures containing *Lactobacillus rhamnosus*, *L. casei*, *L. plantarum*, *L. acidophilus*, *Bifidobacterium longum*, and *B. animalis* subsp. *lactis* in rats with dextran sulfate sodium (DSS)-induced colitis compared to control. **Methods**: Rats were randomly assigned to five groups (5 rats/group; n = 25): G1: negative normal control; G2: positive control (DSS); G3: DSS with sulfasalazine (DSS-Z); G4: DSS with soymilk (DSS-SM), and G5: DSS with fermented soymilk (DSS-FSM). Parameters monitored included the following: the disease activity index (DAI), macroscopic and histological assessments of colitis, and a fecal microbial analysis performed to assess the severity of inflammation and ulceration. **Results**: The DSS-FSM rats group exhibited lower DAI scores (*p* < 0.05) than other treated groups during the induction period. A macroscopical examination revealed no ulceration or swelling in the intestinal mucosa of rats in the DSS-FSM-treated group, resembling the findings in the negative control group. In the positive control (DSS group), the colon tissue showed increased inflammation (*p* < 0.05), whereas those in the DSS-SM- and DSS-FSM-treated rats groups did not show significant macroscopic scores of colitis. The positive DSS control and DSS-Z groups had crypt erosion and ulceration areas, severe crypt damage, and epithelial surface erosion, which were absent in the negative control and DSS-FSM groups. The counts of *Lactobacillus* spp. and *Bifidobacterium* spp. remained stable in both G1 and G5 over 4 weeks. The consumption of fermented soymilk with a mixture of probiotics could minimize the severity of DSS-induced colitis in rats. **Conclusion**, it was found that fermented soymilk containing *Lactobacilli* and *Bifidobacterium* might be an effective vehicle for reducing the severity of DSS-induced colitis in rats.

## 1. Introduction

Many people worldwide suffer from inflammatory bowel disease (IBD). This disease not only affects their health, but has a major impact on their economic situation, psychological state, and work activities [1]. It is part of the spectrum of chronic and recurrent gastrointestinal diseases, including Crohn’s disease (CD) and ulcerative colitis (UC). The UC usually affects the mucosa and submucosa of the colon and is characterized by rectal and colonic mucosal inflammation [2,3]. There is no clear explanation for the cause of IBD [4]. However, several theories have been proposed to offer some insight into the possible cause, including gene–environment interactions, dysbiosis, dysfunction of the mucosal barrier, and chronic inflammation associated with dysregulated immune responses to the intestinal microbiota [4,5].

The estimated incidence of pediatric IBD in Asia and the Middle East ranges from 0.5 to 11.4 (per 100,000 person-years), which is relatively less than the comparable values in Northern and Western Europe, 0.5–23 (per 100,000 person-years), and North America, 1.1–15.2 (per 100,000 person-years) [6]. Although the morbidity remains stable in Western countries, the disease burden is high as the prevalence exceeds 0.3% [7]. In the Arab world, the incidence rate of IBD was estimated to be 2.33/100,000 individuals per year for UC and 1.46/100,000 individuals per year for CD [8]. In Saudi Arabia, the IBD incidence rates ranged from 0.32/100,000 to 1.66/100,000 individuals per year [9]. Saudi Arabia has a high prevalence of IBD [8]. According to Al-Fawzan [10], there is a growing burden of IBD, particularly among younger populations. In the Qassim region, for instance, the data showed that, out of 257 IBD patients, 126 had ulcerative colitis (UC) and 131 had Crohn’s disease (CD).

Previous research has shown that gut bacterial populations play a significant role in human health [11]. There is a substantial decrease in microbiota diversity in ulcerative colitis patients, both quantitatively and qualitatively. Additionally, it was found that mucosal inflammation is closely related to losing anaerobic bacteria [12]. It has been recognized that our diet affects our gut microflora composition. Westernized diets are linked to a decreased gut microbial diversity (dysbiosis), which increases the risk of chronic illnesses such as IBD [13], while a high level of microbial diversity is attributed to plant-based diets rich in dietary fiber.

One potential treatment for IBD is gut modulation by probiotics [14]. *Lactobacilli*, *Bifidobacterium*, and *Enterococcus* are bacteria proven to have a protective effect against IBD [15]. This is because they correct gut microbiota imbalances by promoting beneficial bacteria and inhibiting the growth of harmful pathogens [16], thus improving the microecological community. Furthermore, probiotics improve the intestinal mucosal barrier effectiveness by enhancing the production of mucus, tight junction proteins, and antimicrobial peptides, which strengthen the intestinal barrier and prevent inflammation [17]. Studies found that *Lactobacilli* and *Bifidobacterium* strains reduced the excessive inflammation associated with IBD by regulating the host immune response. These probiotics modulate both innate and adaptive immunity by interacting with immune cells, such as dendritic cells and macrophages, and influencing the production of cytokines. [5,18]. In addition, probiotics can increase Short-Chain Fatty Acid (SCFA) production, which may help mitigate the inflammatory processes seen in IBD [19,20,21,22,23]. Zhang et al. [21] found that *Lactobacillus plantarum* inhibited dextran sulphate sodium (DSS)-induced colitis in mice. In addition, Singh et al. [24] reported the protective efficacy of *Bifidobacterium longum* and *Bifidobacterium breve* strains against DSS-induced colonic inflammation. Moreover, Jakubczyk et al. [25] found that probiotics have beneficial effects when consumed in multiple strains, and their metabolic activity greatly influences their characteristics.

Fermented-plant-based diets with probiotics were found to reduce the risk of IBD development in susceptible patients [20,26,27,28]. Several studies reported that the consumption of fermented-plant-based milk significantly reduced pathogenic bacteria such as Enterobacteriaceae and increased the population of *Lactobacillus* spp. that preserve and function as epithelial cells in the colon, enhance the immune system, and reduce the inflammatory response [29,30,31]. Moreover, Lim et al. [32] indicated that fermented soybeans with probiotics (Cheonggukjang) showed protective properties against DSS-induced colitis. Fermented soy whey with probiotic cultures greatly improved the disease activity index (DAI) and colon morphology in a mouse model of DSS-induced colitis [33]. In a previous study in our lab, we found that phenolic and flavonoid contents in soymilk significantly affected the viable cell counts of probiotic *Lactobacilli* and *Bifidobacterium* mixtures during fermentation compared to individual strains [34,35]. Most previous research has concentrated on individual probiotic strains in animal models [21,24,31], leaving a gap in knowledge regarding the combined effects of multiple probiotic strains in plant-based fermentation. With the increasing interest in fermented-plant-based diets and their potential health benefits, it is essential that we address this gap. Additionally, focusing on the combination of various probiotic strains in a plant-based medium, such as soymilk, offers promising avenues for exploration. However, to the best of our knowledge, this is the first study on the impact of fermented soymilk by a mixture of probiotic *Lactobacilli* and *Bifidobacterium* strains on improving inflammation and gut health in rats with dextran sulphate sodium (DSS)-induced colitis. Therefore, this research aims to investigate the impact of fermented soymilk by a mixture of probiotic starter cultures containing *Lactobacillus rhamnosus*, *L. casei*, *L. plantarum*, *L. acidophilus*, *Bifidobacterium longum*, *and B. animalis* subsp. *lactis* on rats with DSS-induced colitis compared to control.

## 2. Materials and Methods

### 2.1. Plant Milk Preparation

Soybeans (*Glycine max* (L.) Merril) were purchased from a local store and the milk was prepared using the wet-method-cooked slurry process as explained by Shori and Al-sulbi, [36] with some modifications. Clean seeds of soybean (100 g) were soaked in distilled water (1:9 *w*/*w*) during the night (16 h) at room temperature and ground three times over 10 min with a grinder. Following that, the slurry was continuously mixed while boiling at 100 °C for 10 min in an electric oven. Subsequently, a 100-mesh screen was used to filter the heated slurry to remove the solid residue from the milk, cooled to 4 °C, and used within 24 h. Plant experimental research followed relevant institutional, national, and international guidelines and legislation.

### 2.2. Preparation of Starter Cultures

Pure strains of *L. rhamnosus* ATCC 7469, *L. plantarum* ATCC 14917, *L. casei* ATCC 393, *L. acidophilus* ATCC 4356, *B. longum* DSM 20219, and *B. animalis* subsp. *lactis* DSM 10140 were purchased from the National Committee of Microbiology at the University of Ain Shams. All probiotic bacteria were kept in storage at −80 °C. Each strain was prepared according to Aboulfazli et al. [37] with minor adjustments. The experiment involved 100 μL of each *Lactobacillus* spp. strain being added to sterile 10 mL aliquots of MRS broth (HiMedia, India) for inoculation. For *Bifidobacterium* spp., 100 μL of each strain was added to sterile 10 mL aliquots of MRS broth and 0.05% L-cysteine hydrochloride before incubated at 37 °C for 48 h. To produce the pre-inoculum cultures, 1% (*v*/*v*) of the activated culture was inoculated to 10 mL aliquots of sterile reconstituted skim milk (RSM), 1% yeast extract, and 2% glucose.

### 2.3. Preparation of Fermented Soymilk

The fermented soymilk was processed according to Zahrani and Shori, [34] with some modifications. Briefly, the bacterial inoculum was replaced by a starter culture containing an equal quantity of *L. rhamnosus* ATCC 7469, *L. casei* ATCC 393, *L. plantarum* ATCC 14917, *L. acidophilus* ATCC 4356, *B. longum* DSM 20219, and *B. lactis* DSM 10,140 (1:1:1:1:1:1). The starter culture (2% *v*/*v*; containing 10^5^ CFU/mL of individual strains) was added to the soymilk and the mixture was incubated at 40 °C for 9 h [38]. Soymilk (placebo) was prepared using similar procedures and stored at 4 °C without bacterial cultures. Fermented soymilk was prepared freshly and stored at 4 °C for one week.

### 2.4. Animals and Experimental Design

Albino male rats of the Wistar strain were obtained from King Abdulaziz University (KAU), Jeddah, Saudi Arabia. Specific Pathogen Free (SPF) rats weighing about 200 g were used. All experiments were conducted according to the standards of the Animal Ethics Committee at KAU. The experimental design was approved by the Unit of Biomedical Ethics Research Committee, Faculty of Medicine, King Abdulaziz University (KAU), Jeddah, Saudi Arabia (Reference No. 626-22). All experiments were performed according to the ARRIVE guidelines and under the supervision of the Animal Care and Use Committee (ACUC) at King Fahd Medical Research Center, Jeddah, Saudi Arabia (Protocol Number: ACUC-22-09-13). The rats used in this study were housed in sterile, controlled environments to ensure optimal conditions. Sterile cages, bedding, and equipment were utilized to minimize the risk of pathogen introduction, while proper ventilation was maintained throughout the facilities. Stringent cleaning protocols were implemented to prevent cross-contamination, with cages and equipment routinely disinfected and sanitized. Regular health evaluations were conducted to monitor the well-being of the rats. They were placed in polypropylene boxes on shelves equipped with an air filtration system [39], and both temperature (23 ± 2 °C) and relative humidity (60–70%) were continuously monitored using sensors. The rats were randomly assigned to five groups (5 rats/group; n = 25) [39]. The rats in group 1 (negative normal control) included healthy rats treated with distilled water only during the colitis-inducing period and treated with 2 mL of distilled water during the oral sample administration period. The rats in group 2 (positive control; DSS) were treated with 4% DSS in water. The rats in group 3 (DSS-Z) were treated with 4% DSS in water and 100 mg/kg sulfasalazine (Z). The rats in group 4 (DSS-SM) were treated with 4% DSS in water and 2 mL of soymilk (SM) without the starter culture (placebo). Lastly, the rats in group 5 (DSS-FSM) were treated with 4% DSS in water and 2 mL of fermented soymilk (FSM) with a starter culture.

### 2.5. Induction of Colitis and Product Administration

Rats were acclimated for 10 days before the experiment [39]. Dosing of SM and FSM was administered daily by gavage in the morning. The procedure was as follows: from 0–7 days, G4 and G5 were supplemented with an equal volume (2 mL per kg/BW/Day) of SM and FSM, respectively, once a day for one week (Table 1). From the 8th day, UC was induced by administering 4% DSS in drinking water for 7 days of the testing period of all groups except for the G1, which received distilled water (Table 1). DSS solution was freshly prepared and replaced every 2 days. From day 15 to day 30, G3 was treated daily by gavage with 100 mg/kg sulfasalazine for 14 days, while G4 and G5 were treated with 2 mL of SM and FSM, respectively, for 16 days.

### 2.6. Assessment of the Disease Activity Index (DAI)

The colitis was chemically induced by administering dextran sulfate sodium (DSS—MP Biomedicals, USA). The DSS (4%) was dissolved in the water provided daily to the rats for 7 days (W2). The disease activity index (DAI) was scored including body weight loss, stool consistency, and rectal bleeding as described by Toumi et al. [40]. In brief, each score was determined as the extent of body weight loss (0: <1%; 1: 1–5%; 2: 5–10%; 3: 10–20%; and 4: >20%), stool consistency (0 = normal; 2 = loose; and 4 = diarrhea), and gross bleeding (0 = normal color; 2 = reddish color; and 4 = bloody stool), and combined and divided by 3 for each rat.

### 2.7. Colon Assessment

After 30 days of the experiment, the animals were euthanized under isoflurane anesthesia. A laparotomy was performed, and the colon and rectum were removed and opened longitudinally for further macroscopic (colitis severity) and histological (colonic injury) analysis.

#### 2.7.1. Macroscopic Assessment of Colitis

Rats were euthanized under isoflurane anesthesia; then, the colon was excised, opened longitudinally, and washed in saline. The large intestine samples from the different experimental groups were photographed for macroscopic analysis. An evaluation of the macroscopic damage (the area of inflammation and the presence or absence of ulcers) was conducted as described by Huang et al. [41]. Based on a semiquantitative scoring system, macroscopic damage was assessed using the following features: 0 (normal), 1 (focal hyperemia and no ulcers), 2 (ulceration without hyperemia or bowel wall thickening), 3 (ulceration with inflammation at one site), 4 (two or more sites of inflammation and ulceration), 5 (major site of damage extending 1 cm along the length of the colon), and 6 (when the area of damage extended 2 cm along the length of the colon, the score increased by 1 for each additional cm of damage). The colon weight/length ratio was calculated based on the collected data using an analytical balance.

#### 2.7.2. Histological Assessment of Colitis

Tissue samples were taken for histologic evaluation by light microscopy. For light microscope assessment, the samples were fixed by immersion in 10% formaldehyde, washed in running water for 24 h, and stored in 70 GL alcohol. Subsequently, the samples were routinely processed and embedded in paraffin. For each animal, three randomly taken tissue sections (5 μm) were mounted on glass slides, stained with hematoxylin and eosin (H&E), and examined using a photomicroscope. Histological scoring was performed as described by Nanda Kumar et al. [42], based on the following: normal mucosa = surface epithelium intact, crypts normal, and no inflammation; healing ulceration = partial crypt and surface epithelial loss with evidence of re-epithelialization, or crypts showing severe degenerative change; and severe ulceration = complete loss of surface and crypt epithelium extending to muscularis mucosa.

### 2.8. Determination of Fecal Microbial Analysis

The composition of *Lactobacilli* and *Bifidobacterium* strains was analyzed after ingestion of the products (W1), after colitis induction (W2), one week after the end of colitis induction (W3), and at the end of the experiment (W4). The feces were collected from the animals’ containment boxes, stored in sterile polypropylene bags, and frozen at −80 °C for later analysis [43]. The analysis of the composition of the fecal microbiota was based on the determination of the population of bacteria belonging to the genera: *Lactobacillus* spp. and *Bifidobacterium* spp. Stool samples were diluted and inoculated in selective culture media, i.e., *Lactobacillus* spp. in MRS agar at 37 °C for 48 h, and *Bifidobacterium* spp. in MRS-LP agar at 37 °C for 72 h, and both were incubated anaerobically.

### 2.9. Statistical Analysis

All data are expressed as means ± standard error of the mean (SE). The data obtained were analyzed for variance (ANOVA) followed by Duncan’s post hoc test and Tukey’s post hoc test for mean comparison. All analyses were performed using the SPSS statistical analysis program version 20.0 (SPSS Inc., Chicago, IL, USA). A significant difference was considered at the level of *p* < 0.05.

## 3. Results

### 3.1. Colitis Disease Activity Index

**Figure 1:**
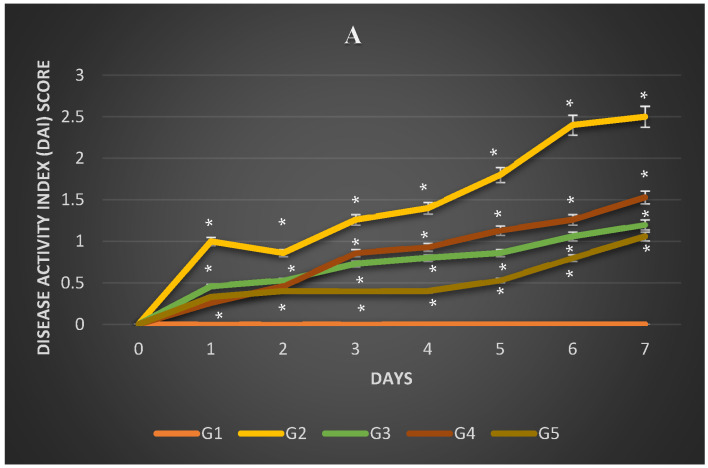

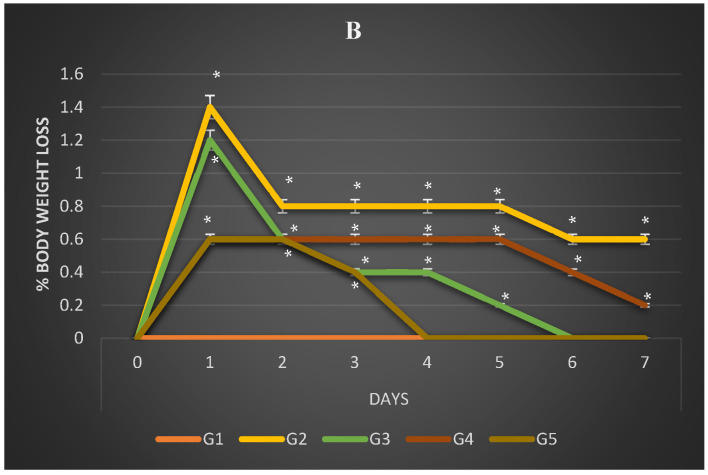
Evaluation of colitis. (**A**) Disease activity index (DAI); and (**B**) body weight change (%) during the induction period in week 2. G1: normal group; G2: DSS group; G3: test groups treated with sulfasalazine; G4: test groups treated with soymilk; and G5: test groups treated with fermented soymilk. Data are presented as mean ± SEM. * The level of significance was present at *p* < 0.05 compared to the control at the same period (5 rats/group; n = 25).

The behavior of the rats in the various groups during the second week after initiation (W2) confirmed that the disease was progressing. The groups of rats (G2, G3, G4, and G5) given DSS showed signs of colitis associated with an increase in the disease activity index (i.e., weight loss, changes in stool consistency, and bleeding) during the induction period (Figure 1A). In the negative control group (G1), the rats did not lose weight, their stool consistency did not change, and no occult blood was observed in their stool during the seven days of induction. On the other hand, the disease activity index (DAI) score in G2 was the highest among other groups from the first day of induction. DAI scores were significantly lower (*p* = 0.000) in rats that consumed fermented soymilk products (G5) compared to the other treated groups (G3 and G4) during the induction period (Figure 1A). Tukey’s post hoc test found that the mean value of DAI scores was significantly different between G1 and G3 (*p* = 0.005), G1 and G4 (*p* = 0.001), G2 and G3 (*p* = 0.005), and G4 and G5 (*p* = 0.000). As a result of treatment with fermented soymilk, rats lost less body weight, had a normal stool consistency until the fourth day, and had occult secretion or apparent blood in their stool only on days 6 and 7. Unlike healthy control rats (G1) that gained weight over 7 days, the body weight of G2 decreased significantly on day 1 (1.4%) up to day 5 (*p* = 0.000; Figure 1B). Rats in G5 showed a significantly reduced body weight after DSS administration on day 4. However, the body weight recovered on day 6 for G3 (Figure 1B). The body weight of G2 continued to decrease until day 7 of the DSS administration. Tukey’s post hoc test found that the mean value of body weight was significantly different between G1 and G4 (*p* = 0.009); G2 and G3 (*p* = 0.040); and G2 and G5 (*p* = 0.002).

### 3.2. Macroscopic Assessment

**Figure 2:**
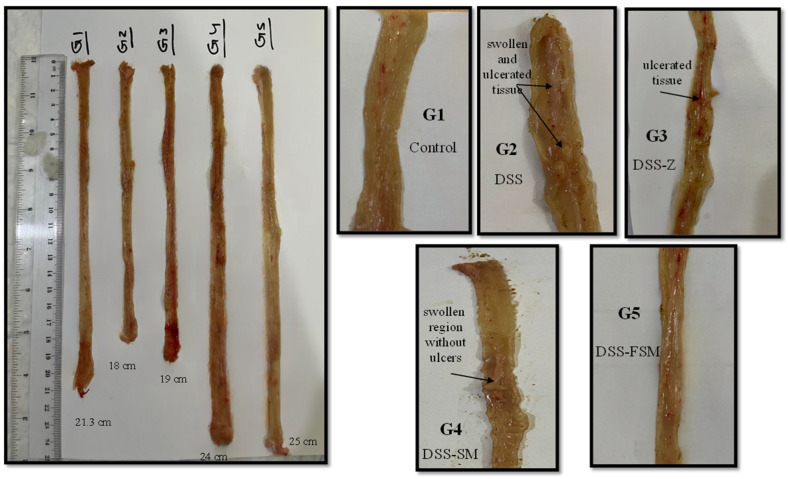
Macroscopic examination of the colons in different groups of rats. G1: normal group; G2: DSS group; G3: test groups treated with sulfasalazine; G4: test groups treated with soymilk; and G5: test groups treated with fermented soymilk.

**Figure 3:**
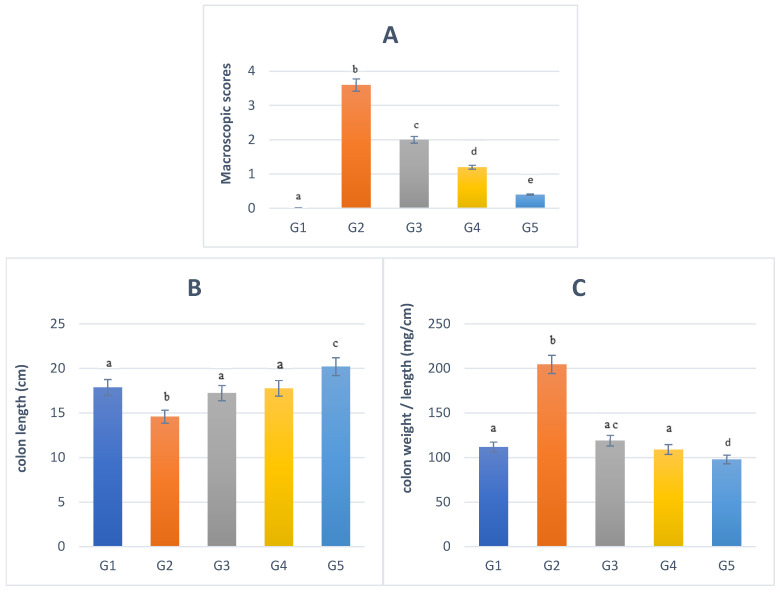
Macroscopic analysis of damage along the colon, which includes macroscopic score (**A**), colon length (**B**), and colon weight/length (**C**). G1: normal group; G2: DSS group; G3: test groups treated with sulfasalazine; G4: test groups treated with soymilk; and G5: test groups treated with fermented soymilk. Data are presented as mean ± SEM. The significance level was present at *p* < 0.05 compared to the control at the same period (5 rats/group; n = 25). ^abcde^ means with different superscript letters indicate the significance level at *p* < 0.05 compared to control.

As illustrated in Figure 2, the macroscopic examination revealed swollen and ulcerated tissue along the intestinal tract of rats in G2 and G3. Furthermore, a swollen region without ulcers was observed in G4. The rats treated with DSS water and fermented soymilk (G5) had no ulceration or swelling of the intestinal mucosa. G5 showed similar results to the control group (G1). Moreover, G2 colon tissues showed an increase in inflammation (*p* = 0.003). In contrast, G3, G4, and G5 colon tissues did not demonstrate significant colitis macroscopic scores (Figure 3A). The colon weight and length of the large intestine were also evaluated to determine the severity of DSS-induced colitis. In G2, the colon length was shorter (14.6 ± 2.81 cm) than in G1 (17.86 ± 2.68 cm; *p* = 0.047: Figure 3B). G5 showed the lengthiest colon (20.2 ± 3.56 cm; *p* = 0.002) among other treatments, whereas G3 and G4 showed an almost similar colon length (~17 cm; *p* = 0.003, *p* = 0.027, respectively). The ratio of the colon weight to colon length (mg/cm) was significantly higher in G2 (204.52 ± 0.21 mg/cm) than in G1 (112.2 ± 0.10 mg/cm; Figure 3C). There was a remarkable difference in the ratio of the colon weight to colon length between G3 (119.25 ± 0.289 mg/cm) and G4 (109.23 ± 0.101 mg/cm) compared with G2 (204.52 ± 0.215 mg/cm, *p* < 0.05), but not with G1 (112.2 ± 0.109 mg/cm, *p* > 0.05). G5 showed a lower weight-to-colon length ratio (98.08 ± 0.09 mg/cm) than other groups.

### 3.3. Histological Assessment

**Figure 4:**
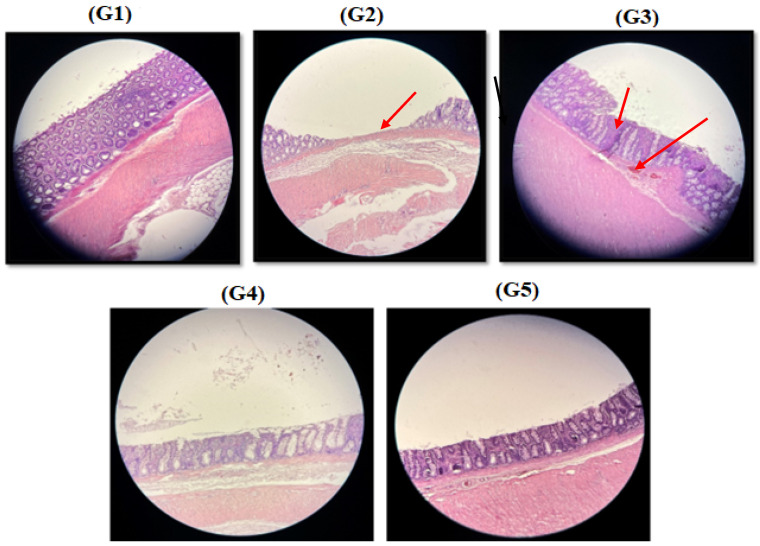
Representative photomicrographs from the different experimental colon groups, stained with hematoxylin/eosin. G1: negative control—healthy epithelium, G2: positive control—crypt erosion (the arrow exhibited epithelium with severe crypt damage), G3: DSS with sulfasalazine—ulceration area (the arrows exhibited epithelium with severe crypt damage), and G4 and G5: DSS with soymilk and DSS with fermented soymilk, respectively—inflammatory cell infiltrated areas, with no crypt alteration.

A significant characteristic of the DSS model is colonic mucosal damage, reflected by increased histological damage severity scores, colonic injury, crypt hyperplasia, and a decreased crypt area. As shown in Figure 4, G2 and G3 had crypt erosion and ulceration areas, severe crypt damage, and epithelial surface erosion, which were absent in G1 and G5. Furthermore, the colon in G4 did not show ulcerations or crypt hyperplasia. A one-way ANOVA revealed a significant difference between the groups (F = 28.444, *p* = 0.000). Tukey’s post hoc test found that the mean value of histological damage significantly differed between G1 and G3–G4 (*p* < 0.005). In addition, G2 showed significant differences with G4 and G5 (*p* = 0.009 and 0.001; respectively). G5 had significant differences with G3 and G4 (*p* = 0.003 and 0.016; respectively).

### 3.4. Fecal Microbial Analysis

**Figure 5:**
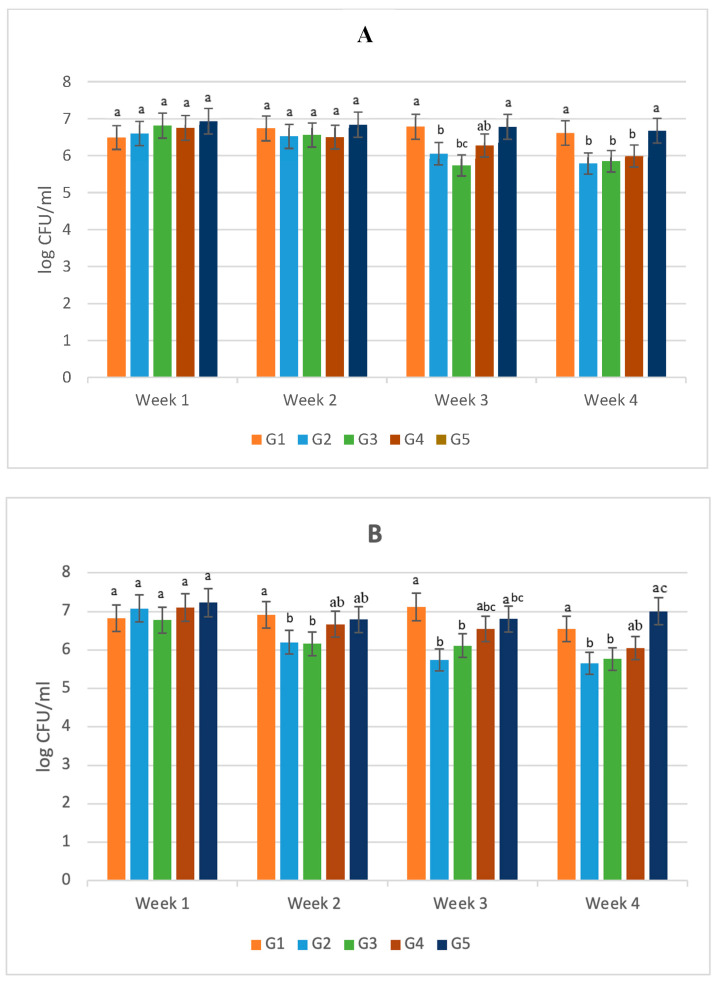
Viable cell counts (VCCs) of *Lactobacillus* spp. (**A**) and *Bifidobacterium* spp. (**B**) among the groups. G1: normal group; G2: DSS group; G3: test groups treated with sulfasalazine; G4: test groups treated with soymilk; and G5: test groups treated with fermented soymilk. Data are presented as mean ± SEM. The significance level was present at *p* < 0.05 compared to the control at the same period (5 rats/group; n = 25). ^abc^ means with different superscript letters indicate the significance level at *p* < 0.05 compared to control.

Figure 5 represents the changes in viable cell counts (VCCs) in the feces of different experimental groups during the protocol. For *Lactobacillus* spp., the negative control group (G1) and fermented soymilk group (G5) maintained a bacterial population of 10^6^ cfu/mL throughout the experiment. However, the other groups that received DSS induction varied in the number of bacterial populations. The variation in the population of *Lactobacillus* spp. in G2 and G3 was less than one log cycle in W4 (Figure 5A). The count of *Lactobacillus* spp. was similar in the control and other groups during W1 and W2. The viable cell counts did not significantly change between G1 and G5 during the experiment. However, the viability in G5 was significantly (*p* < 0.05) higher than in G2 and G3 during W3. In the last week, G5 had the highest viability of *Lactobacillus* spp. (6.679 log cfu/mL) among other treated groups (Figure 5A).

The population of *Bifidobacterium* spp. remained stable in both G1 and G5 throughout the protocol (Figure 5B). There were no significant differences (*p* > 0.05) among the groups for two weeks. However, G2 and G3 had a significant reduction (*p* < 0.05) in *Bifidobacterium* spp. counts compared to G1 and G5 during week 3. G5 showed a significant increase in the population of *Bifidobacterium* spp. (7.006 log cfu/mL; *p* < 0.05) compared to G2, G3, and G4 (5.66–6.05 log cfu/mL) in the last week (Figure 5B).

## 4. Discussion

### 4.1. Colitis Disease Activity Index

The Colitis Disease Activity Index in rats is calculated based on several criteria, including weight loss, stool consistency, and the presence of blood in the stool. Scores are assigned to these factors, which are then used to determine the severity of colitis in rats [43]. Rodents treated with DSS exhibit symptoms similar to those of humans with UC, including bloody diarrhea, weight loss, colon shortening, bloody stools, diarrhea, epithelial damage, and mucosal disruption [43]. In this study, the DSS-treated groups exhibited greater weight loss, and higher DAI scores compared to the normal group. Zhang et al. [44] found that drinking water with DSS caused ulceration and hemorrhagic necrosis in the small intestine and colon, as well as inflammatory cell infiltration. Damage to the small intestinal mucosa leads to nutritional deficiencies, weight loss, and decreased immunity [45]. This result was in agreement with the report by Feng et al. [46] who found significant weight loss in mice given DSS. Furthermore, inflammation in the gut caused by DSS can result in simple occult bleeding [24]. However, fermented soymilk may possess protective properties that could effectively mitigate the severity of inflammation induced by DSS [29,44]. These protective effects may stem from the bioactive compounds in fermented soymilk, such as probiotics and antioxidants, which can enhance gut health, reduce inflammatory markers, and promote mucosal healing [29]. Our findings indicated that fermented soymilk containing different species of *Lactobacilli* and *Bifidobacterium* strains might lower DAI scores and prevent colitis development. These are because of the improved gut barrier health and alleviation of colitis symptoms as a result of *Lactobacilli* and *Bifidobacterium* [34,35,47]. A previous study found that probiotic mixtures could maintain remission in most patients with UC [48]. This is due to increased levels of protective bacteria and further supports the role of probiotics in IBD treatments [49]. Several studies have reported that fermented soy products improved UC and reduced intestinal inflammation in mice fed DSS [43,50], which was consistent with our findings.

### 4.2. Macroscopic Assessment

In the present study, the consumption of fermented soymilk reduced inflammation and ulcerations as well as improved tissue damage in rats with DSS-induced colitis better than sulfasalazine. This could be attributed to the protective effect of fermented soymilk on protecting the intestinal mucosa. Several studies addressed the relationship between fermented soymilk and inflammatory bowel diseases [51,52,53,54]. According to Sadeghi et al. [55], soymilk consumption could benefit IBD patients by increasing the beneficial and commensal bacteria and reducing the mucus-degrading bacteria. Moreover, probiotic bacteria’s interaction with intestinal epithelial cells enhances the epithelial barrier [56]. Kawahara et al. [50] found that fermented soymilk with six strains of lactic acid bacteria (four strains of *Lactobacillus plantarum*, *Lactococcus lactis* subsp. *lactis*, and *Pediococcus pentosaceus*) prevented colon shortening and epithelial cell breakdown in mice fed DSS used for inducing IBD. Furthermore, other studies report that soy products with probiotics may improve macroscopic conditions in DSS-induced colitis [46].

One of the common symptoms of colitis is colon shortening. Colitis usually manifests with edema, and the ratio between the colon weight and length indirectly indicates the extent of the edema (a larger ratio indicates more severe edema) [57]. Therefore, the colon weight/length ratio was used as an essential criterion to identify the severity of ulcerative colitis in rats. This study showed that DSS-induced colitis could cause shorter colons and a higher colon weight-to-length ratio in rats while the group treated with fermented soymilk with *Lactobacilli* and *Bifidobacterium* probiotics could significantly reduce these changes in rats and alleviate colon tissue edema. It has been found that DSS-induced intestinal injury and inflammation can be reduced by microbial metabolites such as unsaturated fatty acids [58,59,60]. Short-chain fatty acids are metabolites produced when gut microbes digest dietary fiber in the colon. They include acetic acid, propionic acid, butyric acid, valeric acid, isobutyric acid, and isovaleric acid. The primary method for reducing intestinal inflammation is protecting the integrity of the intestinal barrier [61]. Some studies have widely demonstrated that SCFAs were able to protect intestinal barrier function [61,62]. Zahrani and Shori, [34,35] reported that fermented soymilk with *Lactobacilli* or *Bifidobacterium* enhanced phenolic compound levels, which may provide antioxidant protection for the intestinal mucosa and prevent the development of UC [63,64,65].

### 4.3. Histological Assessment

Based on the results obtained from this study, DSS caused erosion with a complete loss of surface epithelium because of its direct toxic effect on epithelial cells, resulting in acute colitis [66]. However, the severity post-treatment with fermented soymilk was significantly lower than that observed in G2 and G3. Crypt alterations and ulceration areas were not observed in the epithelium of G4. Fermented soymilk may have attenuated the severity of the chemically induced inflammation caused by DSS. It has been shown that *Lactobacilli* and *Bifidobacterium* species could help prevent and treat intestinal diseases [67]. Moreover, soy-based diet consumption may enhance the growth of lactic acid bacteria, such as *L. plantarum* and *Bifidobacterium* spp., which may be associated with anti-inflammatory and antioxidant properties [35,68,69]. It has been demonstrated that fermented-soy-based products could prevent chemically induced colitis [52,69,70].

Inflammatory cytokine expressions can help in colitis by promoting the recruitment and activation of immune cells to the inflamed area [51]. These cytokines, such as tumor necrosis factor-alpha (TNF-α) and interleukin-6 (IL-6), play a crucial role in initiating and amplifying the inflammatory response, leading to tissue repair and the restoration of intestinal homeostasis [51,52]. Fermented soymilk contains bioactive compounds, such as isoflavones and peptides with anti-inflammatory properties [50,52]. These compounds can help modulate inflammatory cytokine expressions, reducing their levels and mitigating the inflammatory response in rats with DSS-induced colitis [51,52]. Further studies are needed to evaluate the effect of fermented soymilk with probiotic *Lactobacilli* and *Bifidobacterium* strains on inflammatory cytokine expressions, and inflammatory and immune markers.

A previous study found that fermented soymilk with probiotic *Lactobacilli* or *Bifidobacterium* strains possessed antioxidant properties, which may help protect cells from damage [34,35]. These antioxidants could assist in mitigating the inflammatory response in rats with DSS-induced colitis by reducing the oxidative stress and neutralizing the free radicals [47]. Thus, this could help protect cells and tissues from damage, promoting the healing and restoration of intestinal homeostasis. Further study on the impact of fermented soymilk with a mixture of probiotic *Lactobacilli* and *Bifidobacterium* strains on colonic oxidants/antioxidant stress biomarkers in rats with dextran sulfate sodium (DSS)-induced colitis is needed.

### 4.4. Fecal Microbial Analysis

Various studies have shown the interaction between inflammatory bowel disease and the intestinal microbiota [71,72,73]. The disease may arise because of an imbalance in the composition and functions of the microbiota after antibiotic use or dietary changes, which enhances autoimmune disorders and intestinal infections [72,73,74]. Thus, there is a relationship between microbiota communities in the human intestine and inflammatory bowel disease, even though the interaction mechanism between them is unknown [75,76]. IBD is thought to arise from a complex interplay of genetic susceptibility, environmental factors, and microbial interactions [77]. According to studies, patients with IBD have a different gut microbiome than healthy individuals. One significant genetic factor associated with IBD is mutations in the NOD2 gene. The NOD2 gene encodes a protein that is essential for recognizing bacterial components (like muramyl dipeptide) and triggering an appropriate immune response. This protein helps the body identify both pathogenic (disease-causing) and commensal (beneficial) microbes in the gut, ensuring that harmful bacteria are eliminated while beneficial microbes are tolerated [71]. NOD2 deficiency in murine models leads to an altered microbiota, a reduced abundance of ordinarily dominant bacteria such as *Faecalibacterium prausnitzii* [78], and an increased susceptibility to colitis [77,79], highlighting how disruptions in the microbial balance and immune responses contribute to IBD development and progression. Probiotics can correct intestinal microbiota imbalances, promote the microbial community, improve the intestinal mucosal barrier, and decrease gastrointestinal infections [80,81,82,83]. *Lactobacillus* spp. and *Bifidobacterium* spp. have been identified as key probiotics with anti-inflammatory properties and that are potentially protective against IBD [80]. In the present study, G5 had the highest viability of *Lactobacillus* spp. and *Bifidobacterium* spp. in the feces after 4 weeks of treatment. This might be related to various strains of these bacteria present in fermented soymilk. Several factors can influence the viability of LAB in feces after treatment with fermented soymilk. These include the strains used and native bacteria present in the soymilk, the composition of the soymilk itself, the quality of the soybeans, and the individual composition of gut microbiota [11,22,23]. Moreover, competitive interactions or symbiotic relationships with resident microbes can affect the survival of probiotics [22]. A previous study demonstrated that rats ingesting fermented soy products enhanced the growth of *Lactobacillus* spp. by 0.45 log CFU/g [84]. Several studies found that animals who ingested fermented soy products had higher *Bifidobacterium* strains [85,86]. This suggested that the fermentation process produced specific metabolites that could impact the viability of lactic acid bacteria and beneficially modulate the fecal microbiota [85,86]. Fermentation enhances the viability of lactic acid bacteria (LAB) by creating a nutrient-rich environment that supports their growth [87]. The presence of fermentable sugars, proteins, and isoflavones in soymilk allows LAB to thrive and produce beneficial metabolites [88]. The fermentation process of soymilk generates various metabolites, such as organic acids (e.g., lactic acid), which help lower the pH of the gut, inhibiting the growth of harmful bacteria and creating a more favorable environment for beneficial microbes [89]. Additionally, bioactive peptides and isoflavone aglycones can exert antioxidant and anti-inflammatory effects, significantly enhancing the viability of LAB and positively modulating the gut microbiota [90]. However, a study conducted by Wu et al. [86] found that mice with DSS-induced colitis had a significantly lower number of *Lactobacillus* spp. than normal mice. Similarly, it has been reported that the *Bifidobacterium* strain associated with ulcerative colitis rats decreased compared to the control group [91]. This could explain the reduction (*p* < 0.050) in viable cell counts of *Lactobacillus* spp. and *Bifidobacterium* spp. in G2 and G3 in the last two weeks. Further study is needed to analyze the fecal microbiome profile in rats and understand the mechanisms of the relationship between consuming fermented soymilk with a mixture of probiotic *Lactobacilli* and *Bifidobacterium* strains and ulcerative colitis.

## 5. Conclusions

Our study provided a comprehensive approach to obtaining existing knowledge on the biofunctional properties of fermented soymilk containing four species of *Lactobacilli* and two species of *Bifidobacterium* probiotics on rats with DSS-induced colitis. It showed that the consumption of fermented soymilk containing those probiotics could serve as an effective vehicle for reducing the severity of DSS-induced colitis in rats. Treatment with fermented soymilk (G5) significantly reduced DAI scores during the induction period. The macroscopic examination in G5 was similar to that in the negative normal control (G1) in terms of no ulceration and swelling in the intestinal mucosa. G2 (positive control) and G3 (DSS with sulfasalazine) had crypt erosion and ulceration areas, severe crypt damage, and epithelial surface erosion, which were absent in G1 (negative normal control) and G5 (DSS with fermented soymilk). *Lactobacillus* spp. and *Bifidobacterium* spp. maintained viability between 6.5–7 log cfu/mL in the feces of both G1 and G5 over 4 weeks. The consumption of fermented soymilk with a mixture of probiotic *Lactobacilli* and *Bifidobacterium* could minimize the severity of DSS-induced colitis in rats. Nonetheless, this work demonstrated a clear and effective approach to developing synbiotic food with potential healing effects. Further research is needed to investigate the phenolic profile of fermented-soymilk-containing probiotic lactobacilli and Bifidobacterium strains with anti-colitis properties. Additionally, it is important to assess the viability of these cultures during production and storage to ensure sensory acceptability and preserve the nutritional content of the final fermented soymilk. Furthermore, studies should determine whether this product can enhance the immunity of consumers.

## Figures and Tables

**Table 1:** Colitis induction and product administration during the experimental period.

Weeks (W)	Days	Products Administration
W1	0–7 days	G1, G2, and G3: 2 mL of DW
		G4: 2 mL SM
		G5: 2 mL FSM
W2	8–14 days	G1: DW
		G2 and G3: 4% DSS
		G4: 4% DSS + 2 mL SM
		G5: 4% DSS + 2 mL FSM
W3 + W4	15–30 days	G1 and G2: 2 mL of DW
		G3: 100 mg/kg per day sulfasalazine (for 14 day)
		G4: 2 mL SM (for 16 days)
		G5: 2 mL FSM (for 16 days)

SDW: distilled water; SM: soymilk; FSM: fermented soymilk; DSS: dextran sulphate sodium; G1: negative normal control, G2: positive control, G3: DSS-sulfasalazine, G4: DSS-SM, G5: DSS-FSM.

## Data Availability

The datasets used and/or analyzed during the current study are available from the corresponding author upon reasonable request due to privacy concerns.
